# Serum Total Tryptase Level Confirms Itself as a More Reliable Marker of Mast Cells Burden in Mast Cell Leukaemia (Aleukaemic Variant)

**DOI:** 10.1155/2015/737302

**Published:** 2015-02-10

**Authors:** P. Savini, M. Rondoni, G. Poletti, A. Lanzi, O. Quercia, S. Soverini, C. De Benedittis, G. Musardo, G. Martinelli, G. F. Stefanini

**Affiliations:** ^1^Department of Internal Medicine, Faenza Hospital, Viale Stradone 9, 48018 Faenza, Italy; ^2^Department of Clinical Pathology, Centro Servizi Pievesestina, AUSL Romagna, Piazzale della Liberazione 60, Pievesestina, 47522 Cesena, Italy; ^3^Department of Experimental, Diagnostic and Specialty Medicine, Institute of Hematology “L. e A. Seràgnoli”, University of Bologna, Via Massarenti 9, 40138 Bologna, Italy

## Abstract

Mast cell leukemia (MCL) is a very rare form of systemic mastocytosis (SM) with a short median survival of 6 months. We describe a case of a 65-year-old woman with aleukaemic variant of MCL with a very high serum total tryptase level of 2255 *μ*g/L at diagnosis, which occurred following an episode of hypotensive shock. She fulfilled the diagnostic criteria of SM, with a bone marrow smear infiltration of 50–60% of atypical mast cells (MCs). She tested negative for the KIT D816V mutation, without any sign of organ damage (no B- or C-findings) and only few mediator-related symptoms. She was treated with antihistamine alone and then with imatinib for the appearance of anemia. She maintained stable tryptase level and a very indolent clinical course for twenty-two months; then, she suddenly progressed to acute MCL with a serum tryptase level up to 12960 *μ*g/L. The patient died due to haemorrhagic diathesis twenty-four months after diagnosis. This clinical case maybe represents an example of the chronic form of mast cell leukemia, described as unpredictable disease, in which the serum total tryptase level has confirmed itself as a reliable marker of mast cells burden regardless of the presence of other signs or symptoms.

## 1. Introduction

Mastocytosis is a heterogeneous disease characterized by an accumulation of MCs in one or more organs (bone marrow, skin, liver, spleen, gastrointestinal tract, and skeletal system) [1]. MCL is the leukemic variant of SM, defined in the WHO classification by increased numbers of MCs in bone marrow smears (≥20%) and with an amount of circulating MCs >10% of leukocytes in the classical forms or less than 10% in the aleukemic variant of MCL [2–4]. MCL is relatively rare (less than 1% of SM) with a median survival of 6 months only [2, 5–7].

In SM, an elevated serum tryptase level (>20 *μ*g/L) counts as a minor diagnostic criterion as per the WHO framework, but the correlation between tryptase level and MCs burden remains incompletely understood. The former generally reflects the increased burden of MCs in patients with all types of SM and it is the most useful and widely available blood marker to assess changes in the MCs burden in response to cytoreductive therapy [8].

## 2. Case Report

**Figure 1:**
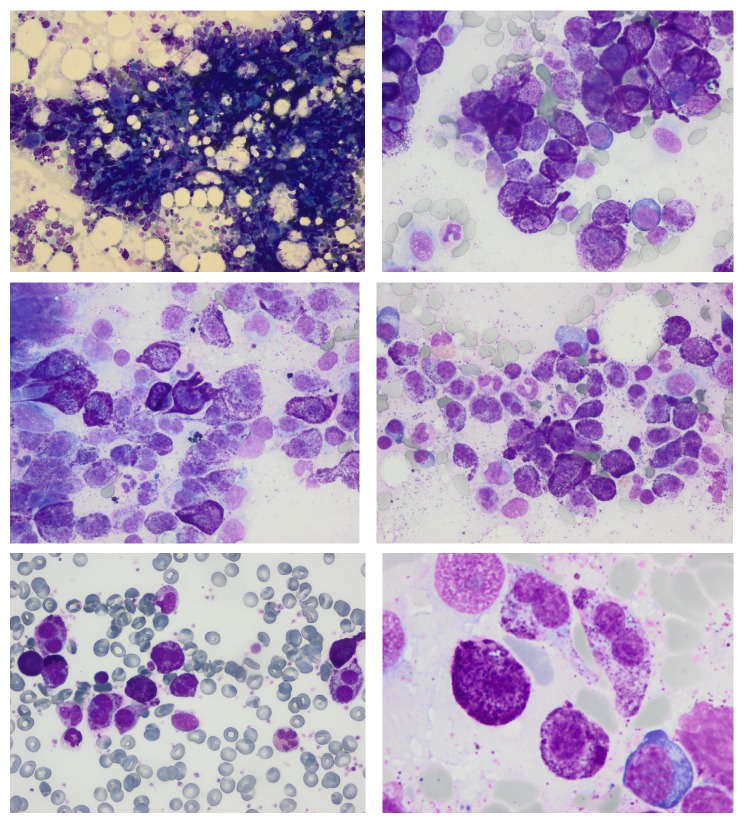
Bone marrow smears at the time of diagnosis (stained by May-Grunwald and Giemsa). Atypical mast cells presented alterations in size and in shape; they presented often with few or numerous metachromatic granules and often contained bilobed nucleus.

**Figure 2:**
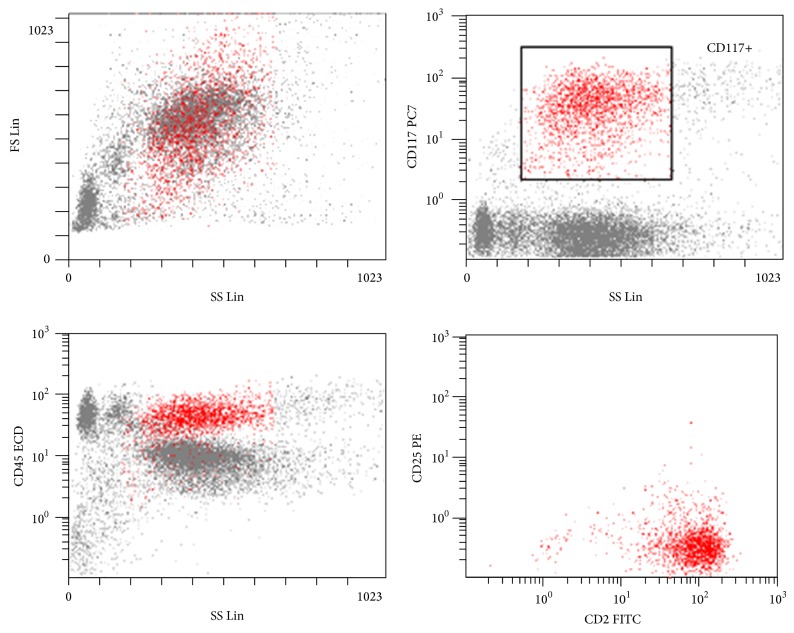
Flow cytometric analysis of expression of CD45, CD2, and CD25 on CD117+ neoplastic mast cells on bone marrow blood at diagnosis.

A 65-year-old Caucasian woman presented to our emergency department because of flushing and hypotensive shock. She did not show serum biochemistry alterations, except for serum total tryptase levels of 2255 *μ*g/L (normal value: <13.0), haemoglobin 10.2 g/dL, total white blood cell count (WBC) 15980 × 10^9^/L, and platelets 319000 × 10^9^/L. She was referred to the hematology unit some days later and the high serum tryptase level was confirmed. She had 50–60% infiltration of atypical MCs on bone marrow smears (Figure 1) and 70–80% on biopsy. Neoplastic MCs showed bright expression of CD117, positivity of CD2, and negativity of CD25 antigen (Figure 2); the morphological examination of peripheral blood smear showed <1% of MCs. Clinical and instrumental examinations excluded organomegaly and any signs of organ impairment; the skin was free of maculopapular lesions, so she was diagnosed as aleukemic variant of MCL. Direct sequencing of the entire KIT gene on bone marrow and peripheral blood tested negative for the KIT D816V mutation and for any other mutation (primers are reported in Table 1).

Due to the asymptomatic presentation, she was given antihistamine alone for five months. Then, the appearance of anemia (9.7 g/dL), with serum tryptase level steadily around 2000 ng/mL, prompted starting therapy with imatinib 400 mg/die—quickly reduced to 300 mg/die due to generalized edema (grade 3 CTCAE v4.0). During the twelve months of imatinib therapy, the patient maintained a stable disease (mild anemia, serum tryptase level of 2000–3000 *μ*g/L, and only few episodes of flushing, abdominal pain, and hypotension, grades 2-3) [9]. Then, imatinib had to be stopped due to increasing anemia and no substantial response. The bone marrow smears at the end of therapy showed a MCs infiltration of 80–90%, with conserved haematological parameters. Over the subsequent 2 months, a rapid progression of mediator-related symptoms was observed along with vertebral osteolysis, increasing pancytopenia, haemoglobin 6.5 g/dL, total WBC 13270 × 10^9^/L, platelets 41000 × 10^9^/L, and serum tryptase levels increase from 4730 to 12960 *μ*g/L. Peripheral blood smears showed 6% of MCs.

The patient refused chemotherapy. Dasatinib therapy 100 mg/die was started while waiting to obtain midostaurin for compassionate use. Unfortunately, the patient died due to haemorrhagic stroke in disseminated intravascular coagulation, three weeks after dasatinib initiation and twenty-four months after initial symptoms.

## 3. Discussion

Chronic forms of MCL have recently been described by Valent et al. [2]. This case perfectly reflects what is described in the literature: a long chronic phase (twenty-two months) and a rapid and fatal evolution of the disease in acute MCL. There are no known factors to predict the evolution and no correlation with tryptase level. This case presented at the beginning with only several allergic symptoms, but with a bone marrow picture of MCL. The medical approach to the majority of mastocytosis patients requires multidisciplinary competence, for the prevalence of allergic symptoms in a hematologic disease. Few drugs are currently approved in the treatment of SM, and imatinib was preferred in our case for the almost complete absence of symptoms and for the absence of any KIT gene mutation. For the second line therapy, midostaurin was chosen for patient refusal to undergo chemotherapy and for substantially stable disease. Serum tryptase level was the only parameter indicating from the beginning an aggressive course of disease. However, the rarity of the disease and the controversial data reported on the role of serum tryptase level as marker of SM burden have to be considered in this setting.

MCL represents an extremely rare disease and chronic forms even more. A recommended clinical approach currently does not exist, so it is very important to share every single clinical experience. In this case, the total serum tryptase level represented a more reliable surrogate marker than any others for evaluating the clinical course of disease. We wonder if we would have had to choose a more aggressive therapy from the beginning based on the very high tryptase level.

## Figures and Tables

**Table 1:** Primer positions according to the reference KIT sequence ENST00000288135 used for sequencing of KIT gene. mT: melting temperature.

Number of PCR	Product length	Sense primer	Position	mT	Antisense primer	Position	mT
1	426	C GAGAGCTGGAACGTGGAC	49	55,9	CCATACAAGGAGCGGTCAACA	474	56,2
2	359	GCACCAACAAACACGGCTTA	387	55,3	CACAGACACAACAGGCACAG	745	55,1
3	391	CCTACCATCGGCTCTGTCTG	630	55,3	ACTACTTCCAAGGTTGTTGTGA	1020	53,6
4	450	TGACTATCAGTTCAGCGAGAGT	921	54,8	TGCCATTCACGAGCCTGTC	1370	55,8
5	392	TCCCAAGTCTGAGAATGAAAGTAAT	1183	54,6	CCTTACATTCAACCGTGCCAT	1574	54,8
6	409	GGAAAGCTAGTGGTTCAGAGTTC	1505	55,4	CCTCAACAACCTTCCCGAAA	1913	53,7
7	385	ACATAGACCCAACACAACTTCC	1806	54,3	GCATGATCTTCCTGCTTTGAAC	2190	54,4
8	393	GCCCACCCTGGTCATTACA	2089	54,6	GCTGCCAAGTCTCTGTGAATAC	2481	55,5
9	393	CCTAGACTTAGAAGACTTGCTGAG	2386	54,7	TGTTCAGGGCTGAGCATCC	2778	55,0
10	420	GGTCGATTCTAAGTTCTACAAGATGA	2719	55,4	GGTGCCCACTATCCTGGAG	3138	54,5
